# AbFlex: designing antibody complementarity determining regions with flexible CDR definition

**DOI:** 10.1093/bioinformatics/btae122

**Published:** 2024-03-06

**Authors:** Woosung Jeon, Dongsup Kim

**Affiliations:** Department of Bio and Brain Engineering, Korea Advanced Institute of Science and Technology, 291 Daehak-ro, Yuseong-gu, Daejeon 34141, Republic of Korea; Department of Bio and Brain Engineering, Korea Advanced Institute of Science and Technology, 291 Daehak-ro, Yuseong-gu, Daejeon 34141, Republic of Korea

## Abstract

**Motivation:**

Antibodies are proteins that the immune system produces in response to foreign pathogens. Designing antibodies that specifically bind to antigens is a key step in developing antibody therapeutics. The complementarity determining regions (CDRs) of the antibody are mainly responsible for binding to the target antigen, and therefore must be designed to recognize the antigen.

**Results:**

We develop an antibody design model, AbFlex, that exhibits state-of-the-art performance in terms of structure prediction accuracy and amino acid recovery rate. Furthermore, >38% of newly designed antibody models are estimated to have better binding energies for their antigens than wild types. The effectiveness of the model is attributed to two different strategies that are developed to overcome the difficulty associated with the scarcity of antibody–antigen complex structure data. One strategy is to use an equivariant graph neural network model that is more data-efficient. More importantly, a new data augmentation strategy based on the flexible definition of CDRs significantly increases the performance of the CDR prediction model.

**Availability and implementation:**

The source code and implementation are available at https://github.com/wsjeon92/AbFlex.

## 1 Introduction

An antibody is an immunoglobulin protein secreted from B cells. It explicitly recognizes foreign molecules called antigens and triggers our body's immune response. An antibody is shaped like a character “Y,” and at the ends of two arms of Y shape, there are loop structures called complementarity determining regions (CDRs), which are the part that complementarily binds to antigens. An antibody usually comprises two protein chains: a heavy chain and a light chain (Murzin *et al.* 1995, Chothia *et al.* 1998). Each chain has three CDRs: HCDR1, HCDR2, and HCDR3 for the heavy chain and LCDR1, LCDR2, and LCDR3 for the light chain. Among them, HCDR3 has a larger sequence variety than the other types of CDR (Xu and Davis 2000), and is more likely to bind complementarily to the antigen, while the other types of CDRs have relatively small variance so that they are clustered into several canonical conformations (Chothia and Lesk 1987, Al-Lazikani *et al.* 1997, Adolf-Bryfogle *et al.* 2015).

Because different CDR sequences produce different antigen-binding surfaces, antibodies can bind to different antigens in a specific manner (Rosenberg and Goldblum 2006, Lippow and Tidor 2007, Karanicolas and Kuhlman 2009). Therefore, a rational design of CDR structures and sequences, especially HCDR3, is important for developing antibody therapeutics that target a specific antigen (Kuroda *et al.* 2012, Sela-Culang *et al.* 2013, Norman *et al.* 2019). In antigen-specific CDR design tasks, one attempts to generate both structures and sequences of CDRs conditioned on a particular antigen, given the antigen–antibody complex structure. Recently, numerous studies have been conducted to design CDRs utilizing various deep learning techniques. Ever since the advent of AlphaFold (Jumper *et al.* 2021), the accuracy of predicting CDR structures from their sequences has increased by deep learning-based models (Ruffolo *et al.* 2020, Abanades *et al.* 2022, Ruffolo *et al.* 2022). Additionally, many deep learning models capable of generating not only CDR structures but also sequences have been proposed (Jin *et al.* 2021, Saka *et al.* 2021, Akbar *et al.* 2022, Eguchi *et al.* 2022, Jin *et al.* 2022, Kong *et al.* 2022, Luo *et al.* 2022).

One of the challenges in developing a deep learning model for antibody design is that there is not enough data for antibody–antigen complex structures (Norman *et al.* 2019) to train the model. Therefore, it is necessary to develop a CDR design model capable of learning 3D structures and sequences in a data-efficient manner. One way to overcome this challenge is to design a deep learning model architecture that exploits the invariant nature of 3D antibody–antigen complex structures under the translational and rotational transformations by using the equivariant neural networks, such as E(n) equivariant graph neural network (EGNN) (Satorras *et al.* 2021), which are known to be more data efficiently. The other way is to artificially expand the training data by adopting an appropriate data augmentation scheme. By effectively employing both approaches, we were able to develop the state-of-the-art antibody design model, AbFlex.

For antibody studies, many different antibody numbering schemes have been developed. The most commonly used numbering schemes include IMGT (Lefranc 1999) and Chothia (Chothia and Lesk 1987) schemes. The largest difference between those numbering schemes is where CDR residues' start and end positions are located. Consequently, CDR residues may differ depending on the numbering scheme chosen, even for the same antibody. To our knowledge, previous studies have used only one particular definition of CDRs, either IMGT or Chothia schemes, for training their models. This could, however, result in a bias in the model. For example, when the prediction models are trained on the dataset created using the Chothia numbering scheme, the models may not perform well for the IMGT scheme-based data.

**Figure 1.**
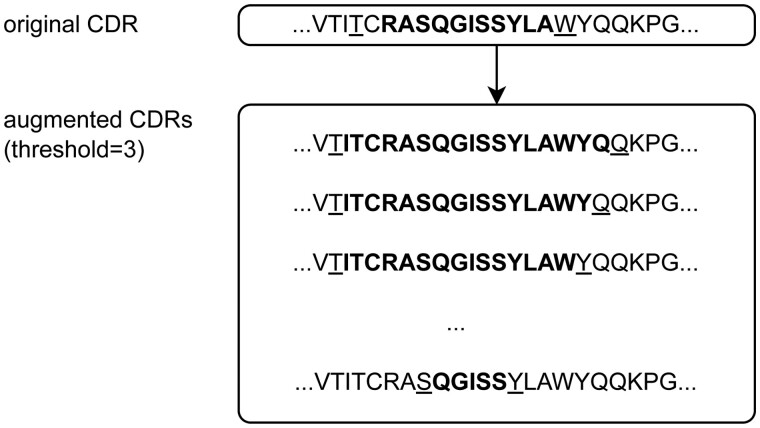
Example of CDR augmentation. The original CDR sequence is highlighted as bold and the anchor residues are indicated with an underline in the upper panel. The augmented CDRs are created by changing the two anchor residue positions within ±*k*, where *k* is a certain threshold value (in this example, *k* = 3). For a single original CDR, as many as (2*k* + 1)^2^ sequences can be created. In this example, there are (6 + 1)^2^ = 49 augmented CDR sequences.

On the other hand, this flexibility in CDR definition provides us an opportunity to develop an effective data augmentation strategy. By flexibly adjusting the anchor residue positions of CDRs, we can create many different sequences with different sizes for a single CDR. As shown in Fig. 1, the augmented CDRs are created by changing the two anchor residue positions within ±*k* residues from the original anchor positions according to a specific numbering scheme (in this study, Chothia scheme), where k is a certain threshold value (in this study, *k* = 5). For a single original CDR, as many as (2*k* + 1)2 sequences can be created. We found that, along with the equivariant graph neural network model, this data augmentation strategy significantly increased the performance of our design model.

In this work, we developed AbFlex, a new antibody design model trained on augmented data using flexible CDR definition. AbFlex can predict the structures and sequences of CDRs simultaneously for a given antibody–antigen complex structure, with state-of-the-art performance in terms of structure prediction accuracy and amino acid recovery rate. Furthermore, AbFlex not only reproduces the correct structures and sequences of antibodies accurately but also generates new antibody sequences estimated to have better binding affinity for their antigens than wild types. As a case study, we illustrate the practical application of AbFlex to redesign HCDR3 of an antibody for binding to a SARS-CoV-2 spike protein.

## 2 Materials and methods

**Figure 2.**
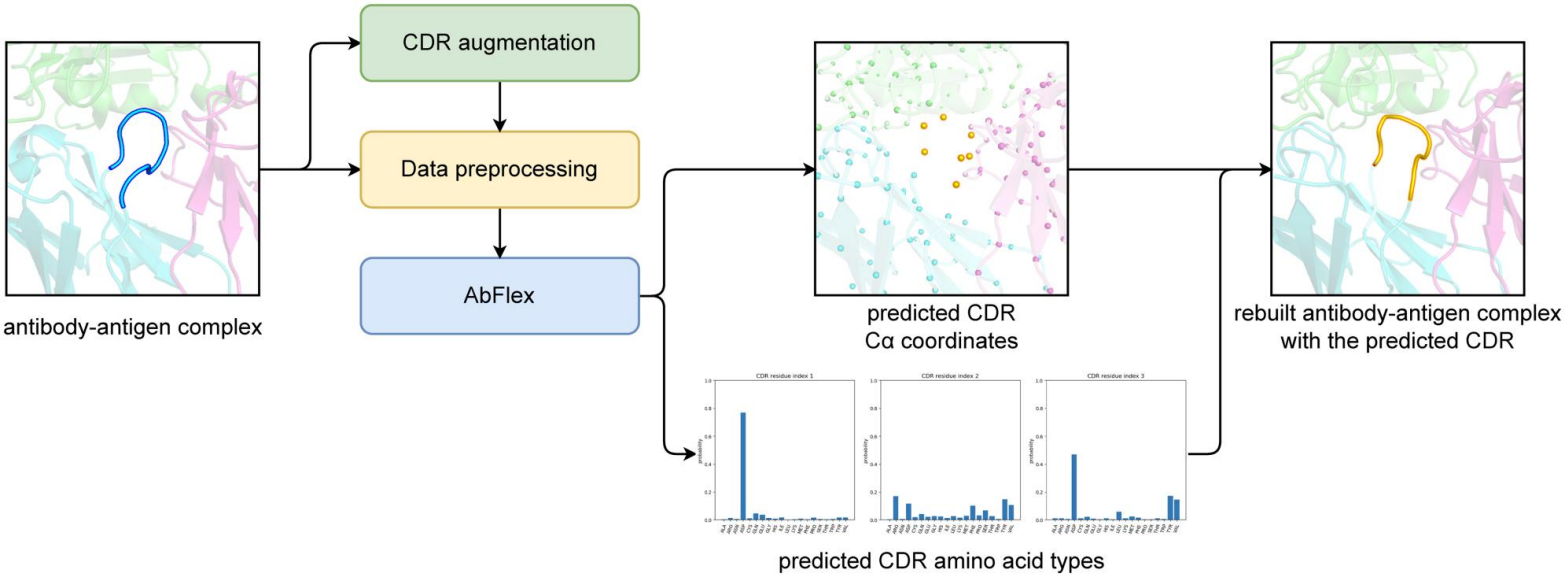
Schematic workflow of AbFlex. The antibody–antigen complex structure data are fed into AbFlex through data augmentation and preprocessing. The CDR augmentation strategy augments the CDR residues in the training set (highlighted as a foreground loop). AbFlex predicts the structures and the amino acid types of the masked CDR residues (highlighted as foreground dots and a foreground loop). It is also able to build a full-atom antibody structure from the predicted results.

A schematic workflow of our antibody design method is shown in Fig. 2. From the antibody–antigen complex structures, the training dataset was created where the CDRs were augmented by the CDR augmentation procedure. During data preprocessing, each antibody–antigen complex structure in the training set was converted into a graph, and its CDR residues to be designed were masked. In a graph representing a protein structure, structures and sequences of the protein were replaced by the node attributes consisting of 3D coordinates of residue Cα atoms and node features. AbFlex was then trained to predict both the structure and sequence of each CDR in the training set, one CDR at a time. A full-atom antibody–antigen complex structure model can be built from the predicted results when desired. It is also possible to estimate the binding affinity of the newly designed antibody using the full-atom structure model.

AbFlex was developed based on EGNN (Satorras *et al.* 2021), a graph neural network equivariant to 3D rotational and translational transformations. EGNN produces robust outputs regardless of the position or rotation of the input in 3D space. It is known that EGNN can learn 3D graph data efficiently without data augmentation by 3D transformation of objects (Satorras *et al.* 2021). By representing protein as a graph (Brinda and Vishveshwara 2005), EGNN can handle 3D protein structure and sequence simultaneously. We hypothesized that these characteristics of EGNN would be suitable for learning protein structures, especially where available structural data is scarce. AbFlex only uses Cα atom coordinates to represent the 3D structure of an antibody–antigen complex to reduce computational cost. The predicted amino acid types appear as probabilities of 20 amino acids from which multiple candidate sequences can be generated through sampling. The predicted CDR Cα coordinates and sampled sequences can generate a new antibody, including backbone and side chains, as shown in Fig. 2.

### 2.1 Dataset

We collected the antibody structure data (PDB file format) from the antibody database, SAbDab (Dunbar *et al.* 2013). From SabDab, data were filtered by antibody–antigen complexes having complete CDR residues (no missing residues in a PDB file) and protein-type antigens. The CDRs of the collected data were defined by the Chothia numbering scheme. The training data having identical CDR sequences to the test set CDRs were removed. Eventually, 32 596 CDR-antigen pairs were generated from 4312 PDB files. The dataset was randomly split by 95:5 for the training and validation sets. We did not remove redundant data with identical CDR sequences within the training set because we desired the model to learn minor structural differences. The test dataset was prepared from the Rosetta AntibodyDesign (Adolf-Bryfogle *et al.* 2018) (RAbD) dataset containing 60 PDBs. The CDRs of the RAbD dataset were defined by both the Chothia and IMGT numbering schemes.

### 2.2 Input

As input, AbFlex takes a graph representing an antibody–antigen complex structure. The input consists of two attributes: the 3D coordinates and the node feature. The coordinates, $x_{\mathrm{input}}\in R^{\left(L,\;3\right)}$, which contain structural information about the antibody–antigen complex, are 3D coordinates of Cα atoms of protein residues, where *L* is the length of the input structure. We used only the Cα atoms to represent a residue-level protein structure in order to reduce the computational cost; required memory size and computation time. The node feature consists of the protein sequence and the chain type. The protein sequence feature is defined as a 256D learnable embedding vector, $\in R^{\left(L,\;256\right)}$, which embeds 20 amino acid types and the special token, <MASK>, for the masked residues. The chain type feature is a 2D learnable embedding vector, $\in R^{\left(L,\;2\right)}$, that distinguishes the residues belonging to the antibody or the antigen. Eventually, the input node feature, $f_{\mathrm{input}}\in R^{\left(L,\;256+2\right)}$, is defined as a concatenation of two features along the axis of the feature dimension.

As we use antibody–antigen complex data, the input includes multiple protein chains of antigens and antibodies. Coordinates and node features from the multiple chains are concatenated along the axis of the chain-length dimension. For example, if an antibody chain has $l_{1}$ residues, and an antigen chain has $l_{2}$ residues, then the shape of input coordinates and features will be $x_{\mathrm{input}}\in R^{\left(l_{1}+l_{2},\;3\right)}$ and $f_{\mathrm{input}}\in R^{\left(l_{1}+l_{2},\;258\right)}$. In addition, to discard the protein chains unrelated to antigen-CDR interactions, we only use antigen residues close to the antibody chain to be designed within a Cα atom distance of 16 Å. Consequently, one input data contains one antibody chain to be designed, its closely located antigen residues, and the counterpart antibody chain (e.g. the light chain for a heavy chain if it exists). Before feeding the input to the model, the coordinates and the sequence of a CDR to be designed are masked. As in ABlooper (Abanades *et al.* 2022), the coordinates of the CDR residues are placed on evenly spaced points along the line connecting two anchor residues. The sequence of CDR residues is masked by <MASK> token.

### 2.3 AbFlex's model architecture

**Figure 3.**
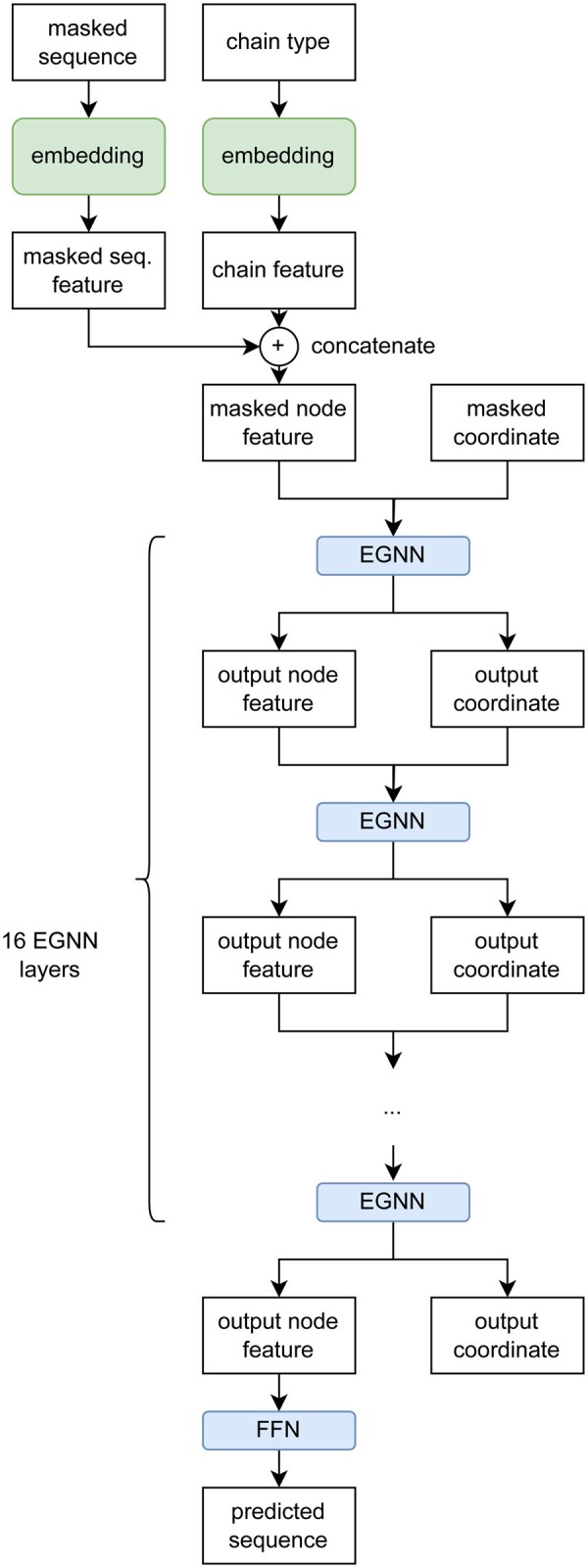
The neural network architecture of the AbFlex. AbFlex consists of 16 EGNN layers and a feed-forward network.

AbFlex consists of 16 sequential EGNN (Satorras *et al.* 2021) layers, as shown in Fig. 3. Each EGNN layer takes the node features and coordinates as input and produces as output the node features and coordinates of the same size as the input. In AbFlex, one node is connected to the 64 nearest nodes forming an edge. A node feature and coordinates are updated through the 64 nearest nodes. Then, the output CDR coordinates are updated using a masking vector, $M\in R^{\left(L,\;1\right)}$, as follows.

$${\tilde{x}}^{k}=x^{k}*M+x_{\mathrm{input}}*\left(1-M\right)$$

(1) $$M\left(i\right)=\left\{\begin{array}{c} 1\;\\ 0\; \end{array}\begin{array}{c} if\;i\in \mathrm{CDR}\;\mathrm{residue}\\ \mathrm{otherwise} \end{array}\right.,\;k\in (1,2,\ldots ,N_{\mathrm{layer}})$$

where $x^{k}$ and ${\tilde{x}}^{k}$ is the output and updated coordinate of the $k^{th}$ EGNN layer, respectively. $N_{\mathrm{layer}}$ is the number of EGNN layers of the model. This operation is done after every EGNN layer to ensure that the residue coordinates, except CDR, remain the same. The multiple EGNN layers update masked coordinates and node features to design appropriate CDR structure and sequence for the antibody–antigen complex. The last output node feature from the last EGNN layer is fed into a shallow feedforward network to produce 20D likelihood logits for 20 amino acid types. The feedforward network consists of two fully connected layers with a nonlinear activation function in between. The detailed hyperparameters can be found at https://github.com/wsjeon92/AbFlex.

### 2.4 Loss design

Since our model predicts CDR structure and sequence, the model uses two types of losses: structure-related and sequence-related losses.

#### 2.4.1 Structure-related losses

We used the root mean squared deviation (RMSD) loss function, $L_{\mathrm{RMSD}}$, as a measure of structural prediction error. Here, only the CDR residues were considered to calculate RMSD. $L_{\mathrm{RMSD}}$ is calculated for all the output coordinates of every EGNN layer.

(2) $$L_{\mathrm{RMSD}}=\frac{1}{N_{\mathrm{layer}}}\sum_{k=1}^{N_{\mathrm{layer}}}\mathrm{RMSD}\left(x_{\mathrm{CDR}}^{k},\;x_{\mathrm{CDR}}^{\mathrm{true}}\right)$$

where $x_{\mathrm{CDR}}^{k}$ is the output coordinates of CDR residues from the $k^{th}$ EGNN layer, and $x_{\mathrm{CDR}}^{\mathrm{true}}$ is the ground truth. The $L_{\mathrm{RMSD}}$ operation is similar to the local loss method suggested by Nøkland and Eidnes (2019)

We also defined the structural violation losses, $L_{\mathrm{repulsion}}$ and $L_{\mathrm{neighbor}}$, which help to prevent the model’s output CDR coordinates from having an unrealistic shape. $L_{\mathrm{repulsion}}$ gives a penalty when each output node coordinate of CDR is within a distance threshold τ of the other non-CDR nodes. The τ value is set to 4.2 Å, determined through statistical analysis of the training data. We first defined the loss for the *k^th^* EGNN layer as follows:

(3) $$L_{\mathrm{repulsion}}^{k}=\frac{1}{N_{\mathrm{CDR}}{*N}_{\mathrm{nonCDR}}}\sum_{i\in \mathrm{CDR},\;j\in \mathrm{nonCDR}}\max(\tau -d_{i,j},0)$$

where $N_{\mathrm{CDR}}$ is the number of CDR residues and $N_{\mathrm{nonCDR}}$ is the number of non-CDR residues. $d_{i,j}$ is a distance between *i*th and *j*th residues. Another structural violation loss $L_{\mathrm{neighbor}}$ is a loss that gives a penalty when the consecutive Cα atom distances of the output CDR coordinates are different from its ground truth. For the *k*th EGNN layer,

(4) $$L_{\mathrm{neighbor}}^{k}=\frac{1}{N_{\mathrm{CDR}}+1}\sum_{i\in \mathrm{CDR}}\left|d_{i-1,\;i}^{\mathrm{pred}}-d_{i-1,i}^{\mathrm{true}}\right|$$

where $d_{i-1,\;i}^{\mathrm{pred}}$ and $d_{i-1,i}^{\mathrm{true}}$ is a distance between (*i* − 1)th and *i*th nodes of a predicted and ground truth structure, respectively. We designed and tested various forms of loss function, and we found that the model performed best when $L_{\mathrm{repulsion}}$ and $L_{\mathrm{neighbor}}$ were applied after the 4th EGNN layer. Therefore, at the beginning of training, the model focuses on reducing the RMSD of the overall structure, and after reasonably good structural prediction has been made (after the fourth layer), structural plausibility measures such as distances between nodes are considered. The overall structure violation losses are given by Equation (5).

$$L_{\mathrm{repulsion}}=\frac{1}{N_{\mathrm{layer}}-4}\sum_{k=5}^{N_{\mathrm{layer}}}L_{\mathrm{repulsion}}^{k},$$

(5) $$\;L_{\mathrm{neighbor}}=\frac{1}{N_{\mathrm{layer}}-4}\sum_{k=5}^{N_{\mathrm{layer}}}L_{\mathrm{neighbor}}^{k}$$

The total structural loss, $L_{\mathrm{structure}}$, is defined as a simple sum of the above three losses.

(6) $$L_{\mathrm{structure}}=L_{\mathrm{RMSD}}+L_{\mathrm{repulsion}}+L_{\mathrm{neighbor}}$$

#### 2.4.2 Sequence-related losses

We used a cross-entropy loss for the CDR sequence prediction. Unlike the coordinates, the node features of all the residues are updated without distinguishing CDR and non-CDR residues after passing each EGNN layer. Therefore, we applied the cross-entropy loss function to both the CDR residues, $L_{CE,\;\mathrm{CDR}}$, and the non-CDR residues, $L_{CE,\;\mathrm{nonCDR}}$ as follows:

(7) $$L_{CE,\;\mathrm{nonCDR}}=\frac{1}{N_{\mathrm{layer}}}\sum_{k=1}^{N_{\mathrm{layer}}}CE(\mathrm{FFN}\left(f_{\mathrm{nonCDR}}^{k}\right),\;{AA}_{\mathrm{nonCDR},\;\mathrm{true}})$$

(8) $$L_{CE,\;\mathrm{CDR}}=\frac{1}{4}\sum_{k=N_{\mathrm{layer}}-3}^{N_{\mathrm{layer}}}CE(\mathrm{FFN}\left(f_{\mathrm{CDR}}^{k+1}\;\right),{AA}_{\mathrm{CDR},\;\mathrm{true}})$$

where the $k^{th}$ output node features of both non-CDR residues, $f_{\mathrm{nonCDR}}^{k+1}$, and CDR residues, $f_{\mathrm{CDR}}^{k+1},$ are converted into 20D logits corresponding to the 20 amino acid types by a simple feedforward network (FFN) and then compared with the true amino acid label of the non-CDR residues (${AA}_{\mathrm{nonCDR},\mathrm{true}})\;$and CDR residues$\;({AA}_{\mathrm{CDR},\mathrm{true}})$.

A structure and a sequence of proteins are interrelated. Therefore, we directed the model to learn sequence prediction ability after the model can reasonably predict the structures. Consequently, $L_{CE,\;\mathrm{CDR}}$ are implemented only for the outputs of the last four EGNN layers. The total cross-entropy loss, $L_{CE}^{\mathrm{total}}$, is defined as a sum of $L_{CE,\;\mathrm{CDR}}$ and $L_{CE,\;\mathrm{nonCDR}}$. To prevent overconfidence in the model, we set label smoothing as 0.1.

#### 2.4.3 Total loss

The total loss of the model is defined as the weighted sum of $L_{\mathrm{structure}}^{\mathrm{total}}$ and $L_{CE}^{\mathrm{total}}$. We gave more weight to $L_{\mathrm{structure}}^{\mathrm{total}}$ than $L_{CE}^{\mathrm{total}}$ to induce the model to learn the structural features first.

(9) $$L^{\mathrm{total}}={10\times \;L}_{\mathrm{structure}}^{\mathrm{total}}+L_{CE}^{\mathrm{total}}$$

### 2.5 Training and evaluation

We trained two models with the same architecture in different ways; one trained with the data of the Chothia numbering scheme, and the other trained with the CDR augmentation. Both models were trained until their performances were saturated. We saved optimal models based on the accuracy of the validation data. The learning rate was set to 1e−6, and an Adam optimizer was used. The models' performance was evaluated by how accurately the trained models reconstructed the CDRs of the test set. We supposed CDRs in an antibody–antigen complex should have optimal structures and sequences to bind the antigen. Therefore, if the predictions of AbFlex are similar to ones with the test set, it implies that AbFlex could design CDR structures and sequences for the target antigens.

Since AbFlex consisted of 16 EGNN layers, there was a gradient vanishing problem despite the residual connection between EGNN layers. To solve this, we implemented loss calculations for not only the last EGNN layer but all the EGNN layers, similar to the local loss technique (Nøkland and Eidnes 2019). Layer-wise training via local loss enables efficient learning even in complex systems. We improved AbFlex's training efficiency and prediction performance by leveraging local loss.

### 2.6 Building a full-atom antibody–antigen complex model from the predicted CDR Cα coordinates and amino acid types

We describe how we build a full-atom antibody–antigen complex model from the predicted CDR Cα coordinates and amino acid types. First, we generate a PDB file of a single antibody chain that contains the designed CDR residues. The designed CDR residues have only the predicted Cα atom coordinates and the corresponding predicted amino acid types. The rest of the antibody atoms remain as the wild-type. The predicted CDR amino acid types are sampled from the probability distributions of the predicted amino acid type for each CDR residue. Then, we merge the generated antibody chain and the rest of the protein chains in the original PDB file. Only the binding chains close to the CDR of design within 16 Å are merged for efficient computation. We use PyMOL (2.4.0 Open-Source) Python library to combine them. Next, we reconstruct the rest of the CDR atoms, including backbone and side chain atoms, using PDBFixer (Eastman *et al.* 2017). PDBFixer cleans up a PDB file that recovers missing atoms and adds hydrogen atoms for a given pH condition. Finally, we relax the complex structure using OpenMM (Eastman *et al.* 2017). OpenMM minimizes the energy potential of the complex structure with the Amber14 forcefield. The rest of the hyperparameters are set as default.

### 2.7 Measuring binding energies

We measure the binding energy between an antibody and an antigen using FoldX AnalyzeComplex (Schymkowitz *et al.* 2005) and Rosetta InterfaceAnalzer (Lewis and Kuhlman 2011, Stranges and Kuhlman 2013). Both methods estimate the binding energy of the complex by calculating energy differences between the bound state and the unbound state of antibodies and antigens. For example, if a complex structure comprises an antibody heavy chain, chain H, light chain, chain L, and antigen, chain A, the binding energy is calculated between the antibody chains and the antigen chain, the interface between HL and A. We relax the complex structure ten times with OpenMM. The relaxed structures are different each time, resulting in different binding affinities. We used the minimum binding energy among the ten relaxed structures. The Rosetta energy unit (REU) was calculated with the REF2015 score function (Alford *et al.* 2017).

## 3 Results

### 3.1 AbFlex performs well regardless of the antibody numbering scheme

We tested AbFlex on the Rosetta AntibodyDesign dataset (RAbD) (Adolf-Bryfogle *et al.* 2018) containing 60 PDBs. We evaluated the model's performance by considering both the structure prediction accuracy and sequence prediction accuracy. The structure prediction accuracy was measured by RMSD of CDR Cα coordinates. The sequence prediction accuracy was measured by CDR's amino acid recovery (Adolf-Bryfogle *et al.* 2018) (AAR), which measures how many predicted amino acid types exactly match their true amino acid types. In “Top1 AAR,” only the most likely amino acid type for each position was considered, while in “Top3 AAR,” if any of the three most likely amino acid types were matched to the true amino acid type, it was accepted as the correct answer. The test sets were prepared with three different CDR number definitions [CDR, IMGT, and North *et al.* (2011)]. North *et al.* define CDRs by clustering structural conformations, and we used PyIgClassify server (Adolf-Bryfogle *et al.* 2015) to define CDRs of the RAbD dataset. AbFlex was evaluated using all three different CDR numbering definitions to examine whether it performed well regardless of the antibody numbering scheme.

The prediction results summarized in Table 1 clearly indicate that we achieved similarly high prediction accuracies on both test sets through the CDR augmentation procedure. All the CDR types except for heavy chain CDR3 (HCDR3) showed sub-Ångstrom RMSD and 54%–80% of AAR in both test sets. The results are explained by the fact that CDR1 and CDR2 have relatively small sequence variety and usually have canonical structures (Chothia and Lesk 1987). Thereby, predicting sequences and structures of CDR1 and CDR2 is considered a relatively easier task than CDR3. While the prediction accuracy of HCDR3 was relatively lower than the other CDR types, our model achieved an RMSD of 1.568 Å and the Top 1 AAR of 37.54% for the IMGT test set, the new state-of-the-art results. In addition, because HCDR3 of IMGT covers a wider range of sequences than HCDR3 of Chothia, it tends to contain a greater number of conserved sequence patterns such as conserved sequences after cysteine. Consequently, sequence prediction of HCDR3 of IMGT tends to have a higher AAR because the conserved patterns are easier to predict. Considering the three most probable amino acid types (Top 3 AAR), the accuracies go up to 48%–88% for all the CDR types.

**Table 1.** Prediction results of AbFlex on RAbD for heavy chain CDRs (HCD1, HCDR2, HCDR3) and light chain CDRs (LCDR1, LCDR2, LCDR3).

		HCDR1	HCDR2	HCDR3	LCDR1	LCDR2	LCDR3
Chothia	RMSD (Å, ↓)	0.603	0.594	1.684	0.351	0.321	0.657
	Top 1 AAR (%, ↑)	69.36	54.44	29.64	80.12	71.88	64.41
	Top 3 AAR (%, ↑)	81.55	69.17	48.09	88.58	84.87	75.93
IMGT	RMSD (Å, ↓)	0.622	0.568	1.568	0.387	0.26	0.657
	Top 1 AAR (%, ↑)	65.99	58.96	37.54	69.82	70.65	64.41
	Top 3 AAR (%, ↑)	79.07	71.90	54.47	81.36	82.99	75.93
North *et al.*	RMSD (Å, ↓)	0.562	0.541	1.579	0.351	0.323	0.657
	Top 1 AAR (%, ↑)	71.66	55.26	37.64	80.12	74.38	64.41
	Top 3 AAR (%, ↑)	82.48	68.67	54.22	88.58	85.58	75.93

### 3.2 AbFlex exhibits the state-of-the-art performance

It is known that the sequences and structures of HCDR3s are the most diverse among all CDRs, and they play a significant role in binding specific antigens. Due to the variety of sequences and structures, predicting the structures and sequences of HCDR3 is a very challenging task. Moreover, it is the most crucial task for antibody design because of its importance in antigen binding. We compared AbFlex's performance for HCDR3 against the other methods. Here we re-trained the AbFlex model with a strict train dataset, which have CDR sequence similarities <50% with the test set (a CDR is defined by Chothia). Eventually, 10349 CDR-antigen pairs (for HCDR3, 2274 pairs were used) were used as the training data and 550 CDR-antigen pairs were used as the validation set. The prediction results are summarized in Table 2, which shows that AbFlex outperforms all the other methods in terms of RMSD and AAR. Compared to the most recent CDR design method, MEAN, the structure prediction accuracy of AbFlex was not significantly different when performing a paired t-test. While the sequence prediction accuracy of AbFlex was significantly (*P*-value <.005) better than ones of MEAN. All the comparison results (RMSD and AAR per PDB id) between AbFlex and MEAN are available in Supplementary Data S1.

**Table 2.** Comparison with other studies for designing HCDR3 of RAbD dataset.^a^

Model	AAR (%, ↑)	RMSD (Å, ↓)
*RosettaAD*	*22.50*	*5.52*
*LSTM*	*22.36*	
*C-LSTM*	*22.18*	
*RefineGNN*	*29.79*	*7.55*
*C-RefineGNN*	*28.90*	*7.21*
*MEAN*	*36.77*	***1.81***
AbFlex	**40.64**	1.88

a The prediction results (AAR and RMSD) of the models in italics are derived from Kong *et al.* (2022).

The results except AbFlex were derived from Kong *et al.* (2022), and the training data for each model did not include any identical CDR to the RAbD dataset. All the CDRs were numbered according to the IMGT scheme. RosettaAD is a conventional CDR design method developed by Adolf-Bryfogle *et al.* (2018) that grafts CDRs from the antibody structure database. Saka *et al.* (2021) and Akbar *et al.* (2022) (LSTM) suggested an LSTM-based CDR sequence prediction model with antibody chains only. C-LSTM is an LSMT model that also utilizes an antibody–antigen complex (Kong *et al.* 2022). RefineGNN by Jin *et al.* (2021) is a graph neural network-based generative model that only predicts CDR structures and sequences autoregressively with antibody chains. C-RefineGNN is a RefineGNN model conditioned to antigen structures. MEAN by Kong *et al.* (2022) predicts CDR structures and sequences conditioned on an antibody–antigen complex by the multi-channel equivariant attention network.

### 3.3 Effect of CDR augmentation

To figure out the effect of the CDR augmentation, we tested our prediction model on the same test set but using the model trained on the dataset prepared by the Chothia scheme only (Table 3). The prediction results on the RAbD dataset prepared by the Chothia scheme were similar to those in Table 1. However, the prediction accuracies for the IMGT and North *et al.*-numbered RAbD dataset became much worse (except LCDR1 and LCDR3, which have identical sequences between Chothia, IMGT, and North *et al.* numbering schemes). These results indicate that the model trained on the training set prepared using a particular CDR definition could produce inconsistent predictions on the test sets prepared using different CDR definitions. Moreover, these results, together with the results shown in Table 1, suggest that antibody design models trained with a single antibody numbering scheme may have been over-trained to that particular training set. We speculate that our data augmentation method improved AbFlex's performance not only by providing more training data but also by helping to avoid over-training.

**Table 3.** Prediction results of AbFlex trained on the training set prepared by the Chothia numbering scheme (without the CDR augmentation).

		HCDR1	HCDR2	HCDR3	LCDR1	LCDR2	LCDR3
Chothia	RMSD (Å, ↓)	0.566	0.552	1.766	0.358	0.343	0.701
	Top 1 AAR (%, ↑)	71.32	58.61	31.56	78.65	73.29	66.12
	Top 3 AAR (%, ↑)	81.51	71.44	46.76	87.20	84.24	75.79
IMGT	RMSD (Å, ↓)	1.935	3.468	3.181	3.007	3.132	0.701
	Top 1 AAR (%, ↑)	40.90	17.51	15.83	14.04	14.41	66.12
	Top 3 AAR (%, ↑)	53.37	34.66	25.71	30.07	29.03	75.79
North *et al.*	RMSD (Å, ↓)	2.284	4.708	3.213	0.358	2.789	0.701
	Top 1 AAR (%, ↑)	20.38	8.59	15.58	78.65	34.65	66.12
	Top 3 AAR (%, ↑)	33.96	21.54	25.47	87.20	48.61	75.79

### 3.4 Binding energy analysis on designed HCDR3 sequences

We compared the predicted binding energies of the antibodies with newly designed HCDR3 sequences to those of the wild types to examine whether our design model could produce novel antibody sequences with improved binding energy. The predicted binding energies of the designed antibodies were measured using FoldX AnalyseComplex (Schymkowitz *et al.* 2005) and Rosetta InterfaceAnalzer (Lewis and Kuhlman 2011, Stranges and Kuhlman 2013). Although predictions of binding affinity using these tools do not completely reflect reality, they are helpful in observing overall trend to some extent. For one antibody–antigen complex structure in the RAbD dataset, we generated ten antibody sequences by sampling ten HCDR3 sequences (sampling in Table 4). We then built full-atom antibody–antigen complex models following the procedure described in the Method section. Also, we generated antibodies with maximum likelihood of sequence prediction(max. likelihood). In addition to the original antibody–antigen complex structures (native), we also built the full-atom models of the wild-type antibodies (reconstructed native) with the CDR Cα coordinates and sequences of the wild-type. In order to achieve an unbiased comparison between the designed antibodies and their wild types, reconstructed native models are needed to correct for the artifacts created during the full-atom building procedure which includes structure relaxation and side chain packing. The binding energies of the sampled antibodies were compared with the native structures and the reconstructed native structures.

**Table 4.** The binding affinity improvement ratio and the success rates of the designed HCDR3s measured by FoldX and InterfaceAnalzer on the RAbD dataset.

	FoldX	Interface Analyzer
Imp. Ratio versus Native (sampling)	18.83%	28.83%
Imp. Ratio versus Reconstructed native (sampling)	38.50%	44.50%
Imp. Ratio versus Native (max. likelihood)	20.00%	23.33%
Imp. Ratio versus Reconstructed native (max. likelihood)	53.33%	41.67%
Success rate versus Native	32/60	42/60
Success rate versus Reconstructed native	51/60	52/60

**Figure 4.**
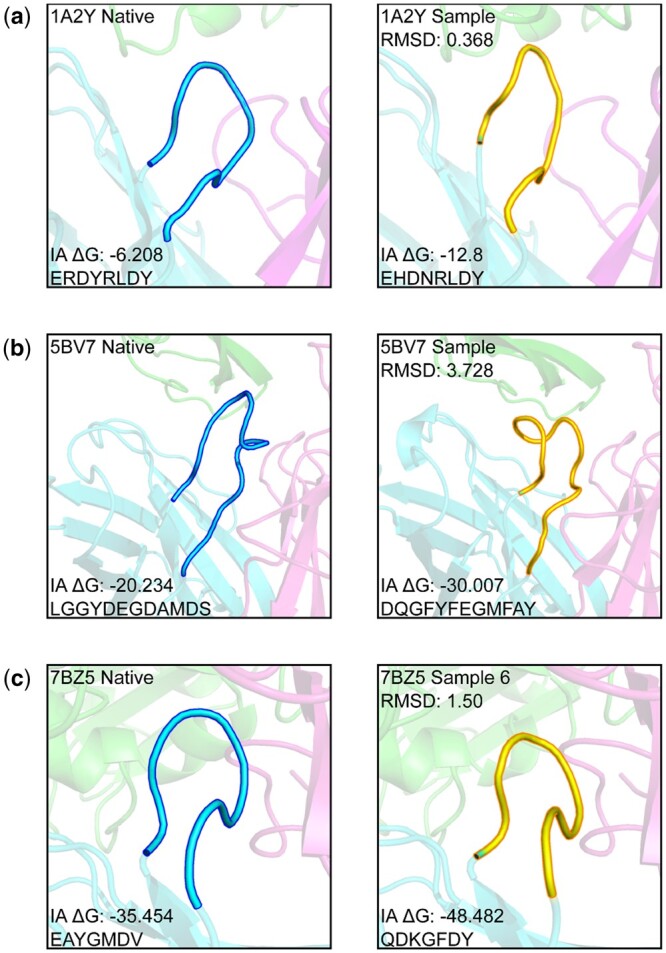
Samples of the designed CDRs compared to their wild types. The designed CDRs are displayed in the right panel of each row. (a) Small RMSD case. (b) Large RMSD case. (c) Redesigned CDR for the SARS-CoV-2 spike protein.

Table 4 shows the improvement ratio of the designed antibodies with lower (better) predicted binding energy than the native (Imp. Ratio versus Native) and the reconstructed native structures (Imp. Ratio versus Reconstructed native). In the case of the designed antibodies with sequence sampling, compared to the natives, the improvement ratio estimated by Foldx and InterfaceAnalyzer was 18.83% and 28.83%, respectively. For reconstructed natives, the improvement ratio estimated by Foldx and InterfaceAnalyzer was 38.5% and 44.50%, respectively. Cases of the designed antibodies with the maximum likelihood sequences are also described in Table 4. Table 4 also shows the success rates. We considered the sequence design a success if at least one out of 10 generated antibodies per one antibody–antigen complex structure had better predicted binding energy. Out of 60 cases in the RAbD dataset, the generated antibodies had better FoldX binding energy than the reconstructed native structures in 51 cases, while in 32 cases, the generated antibodies had better FoldX binding energy than the native structures. Based on InterfaceAnalyzer, 52 and 42 cases had better binding energy than the reconstructed natives and the natives, respectively. Figure 4a and b shows examples of improved predicted binding energy for both small and large RMSD cases. The generated antibody may have a better binding energy than the wild-type antibody despite the predicted structure being quite different from that of the wild-type, as shown in Fig. 4b. This suggests that the predicted CDR structure with a large RMSD may not be a prediction error but a potential CDR structure with a better predicted binding energy. Note that a recently developed model, DiffAb (Luo *et al.* 2022), showed a 23.63% improvement ratio estimated by InterfaceAnalyzer on independent test data of 19 manually selected antibodies.

### 3.5 Case study: SARS-CoV-2 spike protein

Using AbFlex, we redesigned an HCDR3 of the antibody that binds to the SARS-CoV-2 spike receptor-binding domain (PDB ID: 7BZ5), which has been removed from the train set. We masked HCDR3 of 7BZ5 with the Chothia numbering scheme and designed a new structure and sequences using AbFlex. Ten different CDR sequences were sampled, and the full-atom models were built. Finally, the binding energies were estimated by FoldX and InterfaceAnalyzer.

The redesigned structure, sample 6, with a RMSD of 1.50 Å is shown in Fig. 4c, and the newly designed HCDR3 sequences with estimated binding energies are shown in Table 5. According to InterfaceAnalyzer, sample 6 had the best binding energy, −48.482, better than its native or reconstructed native, −35.454 and −34.293, respectively. Sample 4 and 5 was better than the natives for both binding energies. This case study indicates that we can design a potential CDR structure and corresponding sequences that could be expected to bind stronger than the native structure using our CDR prediction model.

**Table 5.** The samples of redesigned sequences of HCDR3 of 7BZ5 and corresponding binding energies compared to its native structures.^a^

	binding energy		HCDR3 sequence
	FoldX (kcal/mol, ↓)	Interface Analyzer (REU^b^, ↓)	
Native	-19.7139	-35.454	EAYGMDV
Reconstructed native	-16.6631	-34.293	
Sample 1	−17.6671	−34.293	HDDGFDY
Sample 2	−17.3002	−22.153	DSDGMDY
Sample 3	−17.0891	−**40.36**	DDYYSDY
Sample 4	−**20.6832**	−**36.364**	GFDGFKY
Sample 5	−**20.991**	−**40.576**	DYDGMDY
Sample 6	−14.8731	−**48.482**	QDKGFDY
Sample 7	−14.142	−34.107	DDVFGDH
Sample 8	−19.5959	−**37.9**	DSDGYDY
Sample 9	−15.5665	−**35.636**	DDDGIDY
Sample 10	−12.7517	−35.183	QRYGKDY

a Bolds are the samples having better binding energies than wild-type models.

b Rosetta Energy Unit.

## 4 Conclusion

In this work, we developed a CDR design model, AbFlex, that predicts the structures and sequences of CDRs for a given antibody–antigen complex. Based on an equivariant graph neural network architecture, AbFlex could efficiently learn CDR structures and sequences despite insufficient antibody structure data. Additionally, AbFlex learned various loop conformations around CDR through the CDR augmentation. As a result, AbFlex's prediction performance became robust regardless of the CDR numbering method. A new data augmentation method appears to have improved AbFlex's performance by providing more training data, in addition to helping to prevent overtraining. In the HCDR3 design task, AbFlex showed state-of-the-art accuracy in terms of structure prediction accuracy and amino acid recovery rate, and >38% of the designed antibodies were estimated to have improved binding energies compared to wild types. In addition, AbFlex can be used to design CDRs whose lengths are different from those of the original CDR by adding or removing residues. To illustrate a practical application of our model, we suggested candidate antibody structures and sequences designed by AbFlex that were expected to bind a SARS-CoV-2 spike protein.

There are several limitations to AbFlex. First, AbFlex predicts only one optimal CDR structure for an antibody–antigen complex, so it cannot generate a variety of structures as many generative models do (Luo *et al.* 2022). Second, when training an AbFlex model, we did not fully consider the binding affinities of antibody and antigen complexes. Although some antibody–antigen complexes may have weaker binding energy than others, we treated all the complexes equally when we trained the model. Third, AbFlex predicts CDRs under the assumption that structural and sequence changes in CDRs do not alter other non-CDR structures of the complex structures. Lastly, AbFlex's prediction accuracy still has room for improvement.

Despite these limitations, AbFlex can be a valuable tool for designing antibodies with improved binding affinity, as we have demonstrated in this work that in >80% of cases, newly designed antibodies were estimated to have better binding energies than wild types in the 60 complexes in RAbD dataset. Through sequence sampling, a large number of antibodies with high binding energies to the target antigen can be generated in a relatively short time. For example, in the case of 7BZ5, generating 100 full-atom antibody models with ten relaxations by openMM and Rosetta InterfaceAnalyzer took around 6.5 h with Nvidia Quadro RTX 5000 GPU and Intel(R) Xeon(R) W-2225 CPU 4.10 GHz. It is also possible to build the full-atom complex structure models for further structural analysis via the provided source code at https://github.com/wsjeon92/AbFlex.

## Supplementary Material

btae122_Supplementary_Data
