# Supplementary material for: AbFlex: designing antibody complementarity determining regions with flexible CDR definition
Source: Bioinformatics. 2024 Mar 6;40(3):btae122. doi: 10.1093/bioinformatics/btae122 (PMC10965422; doi:10.1093/bioinformatics/btae122)
Supplement: btae122_Supplementary_Data [file btae122_supplementary_data.docx]

Supplementary data 1. (a) RMSD and (b) AAR of the RAbD dataset. CDRs are defined by the IMGT definition.

|  | **RMSD (Å)** | | **AAR (%)** | |
| --- | --- | --- | --- | --- |
| **PDB ID** | **AbFlex** | **MEAN** | **AbFlex** | **MEAN** |
| 1a14 | 1.651 | 2.300 | 66.67 | 46.67 |
| 1a2y | 0.526 | 1.113 | 40.00 | 40.00 |
| 1fe8 | 0.728 | 1.130 | 44.44 | 44.44 |
| 1ic7 | 1.233 | 0.864 | 71.43 | 42.86 |
| 1iqd | 1.139 | 1.593 | 30.00 | 30.00 |
| 1n8z | 2.470 | 1.623 | 53.85 | 46.15 |
| 1ncb | 1.786 | 2.157 | 38.46 | 30.77 |
| 1osp | 1.795 | 2.221 | 28.57 | 28.57 |
| 1uj3 | 0.766 | 1.050 | 60.00 | 50.00 |
| 1w72 | 1.570 | 1.748 | 46.67 | 40.00 |
| 2adf | 1.129 | 1.463 | 54.55 | 36.36 |
| 2b2x | 2.666 | 2.170 | 58.33 | 58.33 |
| 2cmr | 1.576 | 1.475 | 41.67 | 33.33 |
| 2dd8 | 1.239 | 1.182 | 45.45 | 36.36 |
| 2ghw | 1.974 | 2.028 | 50.00 | 50.00 |
| 2vxt | 1.474 | 1.546 | 33.33 | 50.00 |
| 2xqy | 0.940 | 0.986 | 36.36 | 54.55 |
| 2xwt | 1.682 | 1.864 | 8.33 | 16.67 |
| 2ypv | 1.837 | 1.600 | 58.33 | 50.00 |
| 3bn9 | 4.174 | 1.828 | 28.57 | 22.22 |
| 3cx5 | 1.630 | 1.287 | 40.00 | 46.67 |
| 3ffd | 1.229 | 1.398 | 36.36 | 45.45 |
| 3h3b | 2.273 | 2.330 | 30.77 | 22.22 |
| 3hi6 | 2.639 | 2.129 | 38.46 | 23.08 |
| 3k2u | 1.964 | 1.372 | 45.45 | 36.36 |
| 3l95 | 2.373 | 1.725 | 41.67 | 50.00 |
| 3mxw | 1.157 | 1.546 | 58.33 | 41.67 |
| 3nid | 2.878 | 2.937 | 25.00 | 41.67 |
| 3o2d | 1.916 | 2.027 | 26.67 | 33.33 |
| 3rkd | 1.617 | 1.699 | 37.50 | 31.25 |
| 3s35 | 1.329 | 0.947 | 50.00 | 50.00 |
| 3uzq | 1.185 | 2.068 | 44.44 | 55.56 |
| 3w9e | 1.763 | 2.285 | 33.33 | 26.67 |
| 4cmh | 1.468 | 1.581 | 46.15 | 38.46 |
| 4dtg | 3.264 | 1.857 | 35.71 | 28.57 |
| 4dvr | 3.073 | 1.890 | 25.00 | 16.67 |
| 4etq | 1.632 | 1.360 | 33.33 | 33.33 |
| 4ffv | 0.850 | 1.106 | 40.00 | 30.00 |
| 4fqj | 2.204 | 3.054 | 61.11 | 50.00 |
| 4g6j | 1.648 | 1.318 | 63.64 | 54.55 |
| 4g6m | 1.559 | 1.784 | 25.00 | 25.00 |
| 4h8w | 3.139 | 1.720 | 58.33 | 33.33 |
| 4ki5 | 1.128 | 3.487 | 44.44 | 40.00 |
| 4lvn | 2.623 | 1.843 | 23.08 | 30.77 |
| 4ot1 | 3.143 | 3.333 | 25.00 | 16.67 |
| 4qci | 1.366 | 1.525 | 30.77 | 30.77 |
| 4xnq | 1.534 | 1.480 | 25.00 | 25.00 |
| 4ydk | 3.717 | 3.761 | 31.82 | 36.36 |
| 5b8c | 1.654 | 1.739 | 38.46 | 46.15 |
| 5bv7 | 2.789 | 2.470 | 27.78 | 36.84 |
| 5d93 | 0.809 | 1.048 | 55.56 | 44.44 |
| 5d96 | 2.421 | 2.171 | 50.00 | 25.00 |
| 5en2 | 3.074 | 3.396 | 47.06 | 47.06 |
| 5f9o | 2.011 | 1.372 | 20.00 | 13.33 |
| 5ggs | 1.545 | 1.794 | 46.15 | 46.15 |
| 5hi4 | 1.439 | 1.436 | 36.36 | 18.18 |
| 5j13 | 2.483 | 1.751 | 33.33 | 26.67 |
| 5l6y | 3.688 | 1.864 | 40.00 | 26.67 |
| 5mes | 1.423 | 1.332 | 41.67 | 33.33 |
| 5nuz | 0.812 | 1.234 | 30.77 | 38.46 |
| **Average** | **1.880** | **1.806** | **40.64** | **36.72** |
| **Std.** | **0.8211** | **0.6422** | **12.84** | **11.21** |
